# Supplementary material for: De Novo Linear Phosphorylation Site Motifs for BCR-ABL Kinase Revealed by Phospho-Proteomics in Yeast
Source: J Proteome Res. 2023 Apr 13;22(6):1790–9. doi: 10.1021/acs.jproteome.2c00795 (PMC10243146; doi:10.1021/acs.jproteome.2c00795)

Supplemental Figure 1

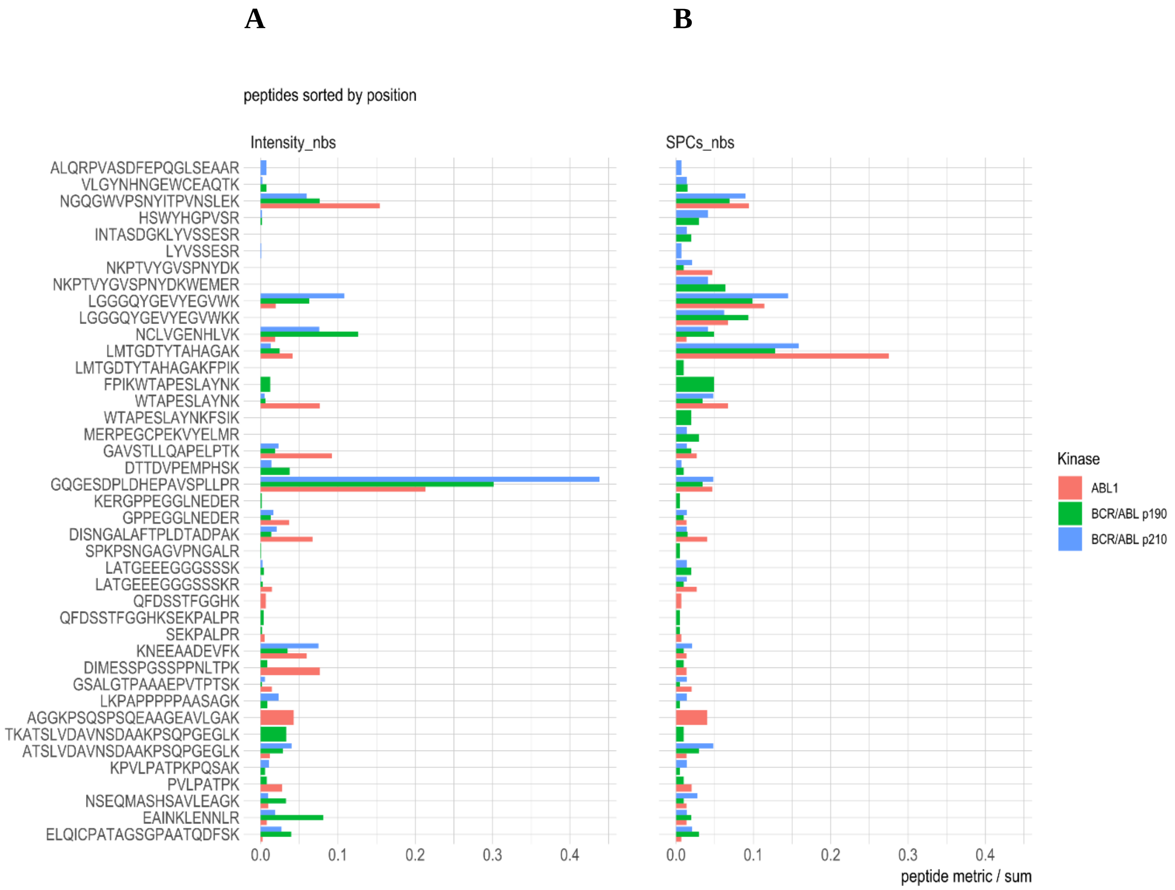

Peptide quantification of human BCR-ABL or ABL1 in yeast.

Tryptic peptides were sorted by position and all peptides that cover identical regions in BCR-ABL and ABL1 are shown. Peptide metrics (**A:** intensity or **B**: SPC) were normalized to the sum of the peptide metric within that kinase (nbs).

Human kinases were expressed at similar levels in yeast.

Supplemental Figure 2

A

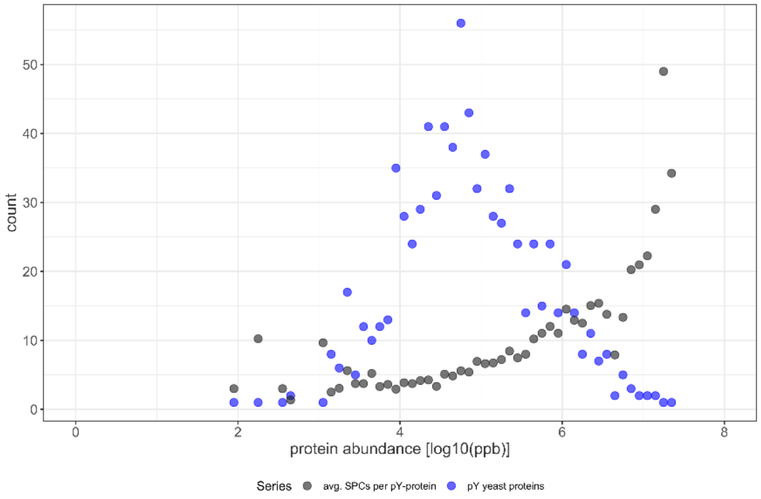

B

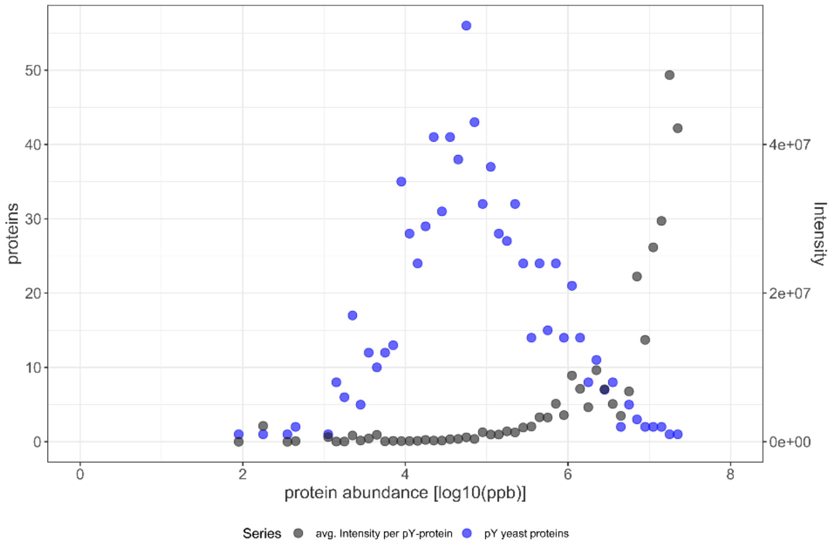

Abundance distribution of pY-yeast proteins.

The X-axis represents yeast protein abundance in log10 parts per billion (ppb) from the Saccharomyces cerevisiae dataset of PaxDB (DOI: 10.1002/pmic.201400441), data are presented as binned (0.1) averages of pY-proteins. Blue points: abundance distribution showing the number of the proteins with pY-sites in our data set in the abundance bin.

A. Gray curve shows SPC count of pY-peptides for proteins in the abundance bin.

B. Gray curve shows average pY-peptide intensity of proteins in the abundance bin (secondary axis).

Quantitative measures of pY-peptides increased with yeast protein abundance.

Supplemental Figure 3

Presentation of images of the ENTIRE membrane in Figure 1

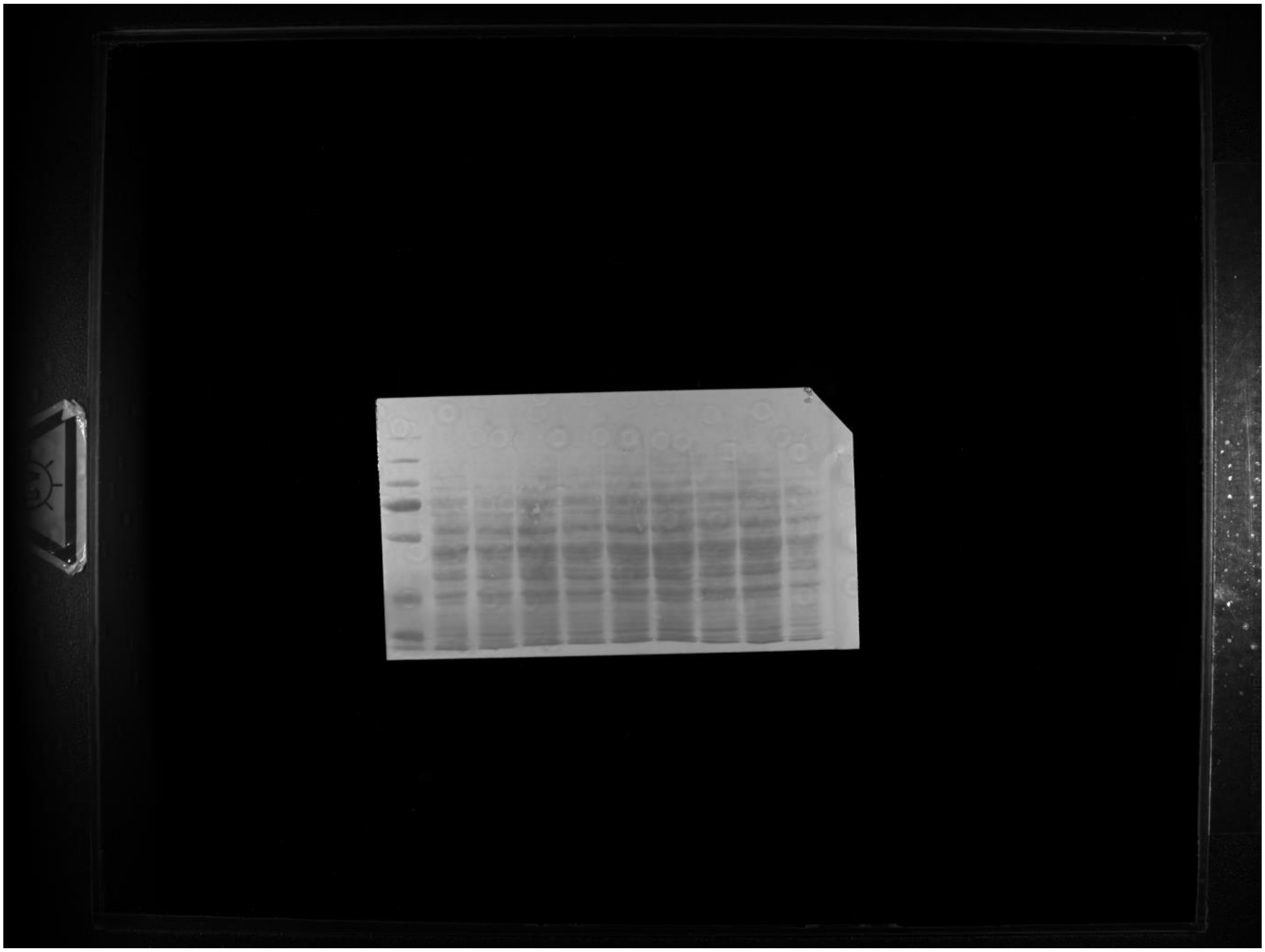

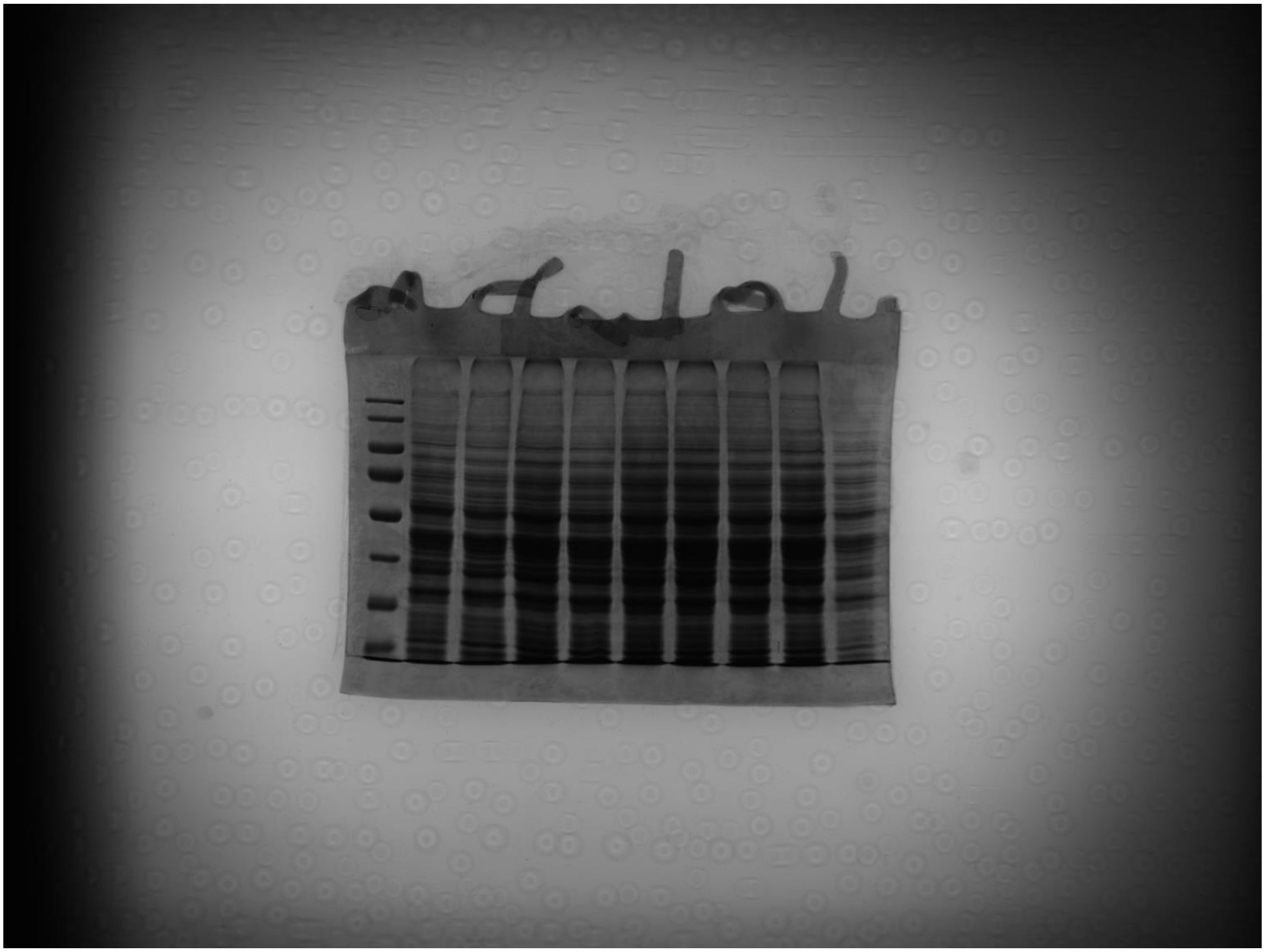

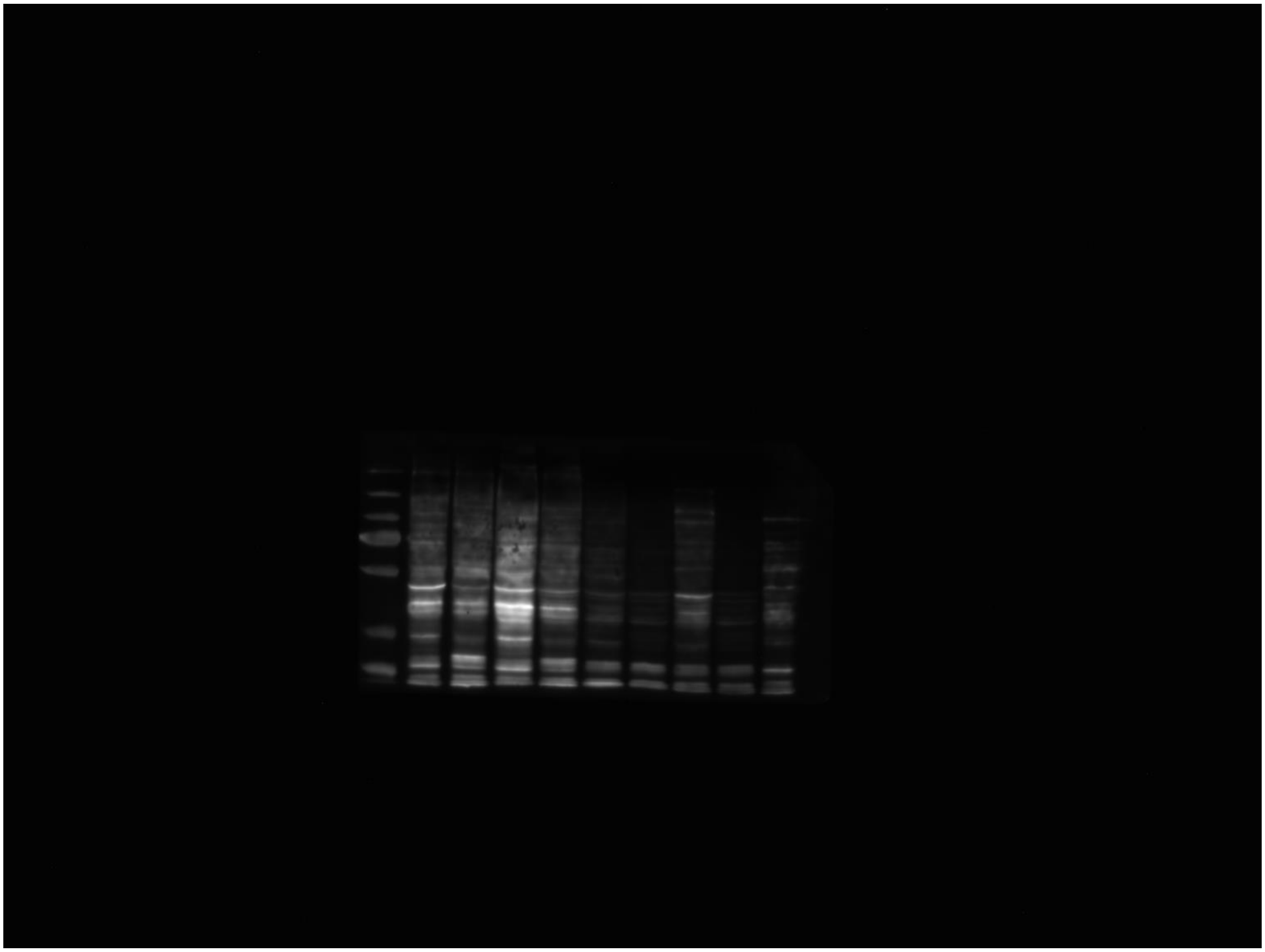

Supplement: Supplementary file 2 — pr2c00795_si_002.pdf [file pr2c00795_si_002.pdf]
